# Spaceflight-induced alternative splicing during seedling development in *Arabidopsis thaliana*

**DOI:** 10.1038/s41526-019-0070-7

**Published:** 2019-04-03

**Authors:** Nicole S. Beisel, Jerald Noble, W. Brad Barbazuk, Anna-Lisa Paul, Robert J. Ferl

**Affiliations:** 1 Plant Molecular & Cellular Biology Program, University of Florida, Gainesville, FL USA; 2 Interdisciplinary Center for Biotechnology Research, University of Florida, Gainesville, FL USA; 3 Department of Biology, University of Florida, Gainesville, FL USA; 4 Genetics Institute, University of Florida, Gainesville, FL USA; 5 Department of Horticultural Sciences, University of Florida, Gainesville, FL USA

## Abstract

Plants grown in spaceflight experience novel environmental signals, including those associated with microgravity and ionizing radiation. Spaceflight triggers a response involving transcriptional re-programming and altered cell morphology, though many aspects of this response remain uncharacterized. We analyzed the spaceflight-induced transcriptome with a focus on genes that undergo alternative splicing to examine differential splicing associated with spaceflight—an unstudied characteristic of the molecular response to spaceflight exposure. RNA sequence data obtained during the APEX03 spaceflight experiment that was collected from two *Arabidopsis thaliana* ecotypes at two seedling stages grown onboard the International Space Station, or as ground controls at Kennedy Space Center, were re-examined to detect alternative splicing differences induced by spaceflight. Presence/absence variation analysis was used to identify putative expression-level differences in alternatively spliced isoforms between spaceflight and ground controls and was followed by analysis of significant differential alternative splicing. This study provides the first evidence of a role for alternative splicing in the molecular processes of physiological adaptation to the spaceflight environment.

## Introduction

During growth on the International Space Station (ISS), plants encounter a suite of abiotic stressors largely owing to the effects of microgravity, cosmic radiation, and other less-characterized stresses associated with the spaceflight environment. Similar to observations made in response to terrestrial plant stresses, exposure to the spaceflight environment triggers a unique transcriptional response.^1–4^ Patterns of spaceflight-induced gene expression have been analyzed in a variety of experiments carried out onboard the ISS, with focus on dissecting the transcriptome at the resolution of individual genes rather than transcript isoforms.^1–4^

Alternative splicing (AS) is the phenomenon by which exons and introns can be differentially joined together to result in the production of multiple distinct transcript isoforms derived from a single parent gene locus (reviewed in ref. ^5^). AS greatly enhances the diversity of the eukaryotic transcriptome. In the model plant *Arabidopsis thaliana*, as many as 60% of intron-containing genes undergo AS.^6^ In addition, unique AS patterns arise in response to terrestrial plant stressors.^5,7–11^ To investigate the role of AS during spaceflight adaptation in plants, RNA sequencing data collected during the APEX03 experiment from *A. thaliana* seedlings grown on board the ISS in comparison to seedlings grown as ground controls at Kennedy Space Center (KSC) were analyzed using a bioinformatics pipeline specifically developed to be aware of AS events (adapted from ref. ^12^).

The APEX03 experiment featured two different *A. thaliana* ecotypes, Columbia-0 (Col-0) and Wassilewskija (WS), and involved RNA sequencing of root tissue from both 4- and 8-day-old seedlings grown on 10 cm^2^ petri plates containing Phytagel then preserved in RNALater on orbit. These time points were chosen because by 8 days of growth *Arabidopsis* seedlings will transition to photosynthesizing using true leaves, which is a major shift during seedling development. Root morphological differences between Col-0 and WS cultivars have been studied in spaceflight previously: Col-0 is known to exhibit much weaker root skewing in microgravity, compared to the strong rightward root skewing observed in the WS ecotype.^13^

**Fig. 1:**
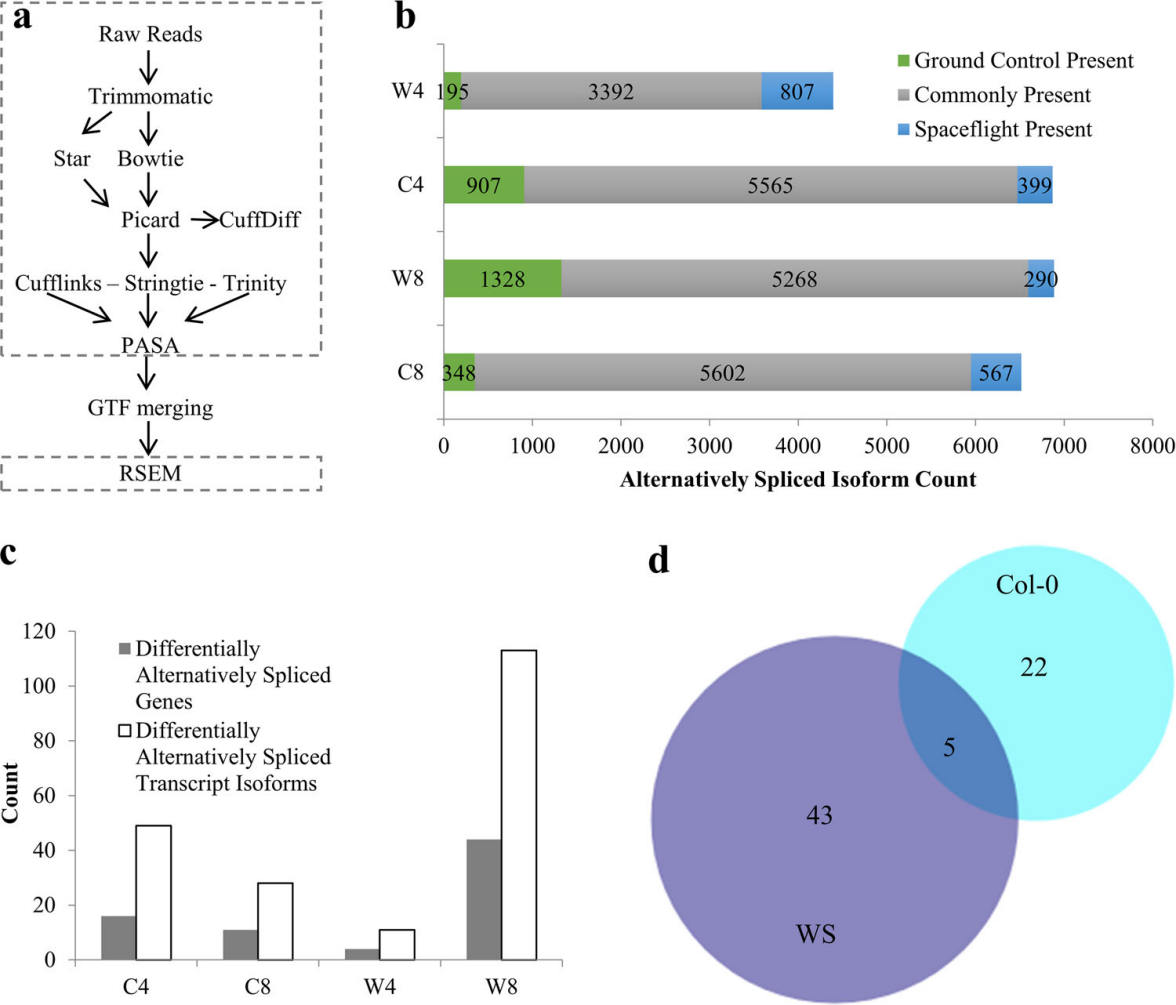
Detection of spaceflight-induced alternatively spliced transcript isoforms. **a** Bioinformatics pipeline utilized to identify all transcript isoforms and significantly differentially spliced genes present in the original RNA sequencing dataset. Steps inside the dashed outline were performed on each individual replicate. See Methods for more detail on gtf merging. **b** Comparison of alternatively spliced isoforms present or absent in spaceflight in both ecotypes and developmental ages tested. **c** Count of differentially alternatively spliced genes and the associated count of transcript isoforms observed in each condition. **d** Comparison of the differentially alternatively spliced genes observed in the Columbia-0 (Col-0) or the Wassilewskija (WS) ecotype background. Differentially alternatively spliced genes observed at both 4- and 8-day time points in either ecotype background were pooled for this analysis. (C Columbia-0, W Wassilewskija, 4 4 days of age, 8 8 days of age. C4 Columbia-0 4 days of age, W8 Wassilewskija 8 days of age)

Following RNA sequencing, the raw reads were processed using a bioinformatics pipeline designed to analyze the transcriptome with resolution at the level of individual transcript isoforms (Fig. 1a) (adapted from ref. ^12^). This pipeline differs from standard pipelines utilized to assess differential gene expression in that three distinct transcript assembly programs are used to maximize the robustness of transcript isoform discovery.^12^ Presence/absence variation (PAV) of AS isoforms was then performed utilizing extremely stringent criteria to identify the expression level differences in AS isoforms between spaceflight and ground controls. To count an AS isoform as present in each condition, the isoform was required to be expressed at an FPKM (fragments per kilobase of transcript per million mapped reads) ≥10 in all four biological replicates of a given condition and was required to come from a parent gene that undergoes AS as identified by the pipeline used in this analysis. By using a high FPKM value as a strict expression level cutoff and eliminating results with biological variation, lists of AS isoforms strongly present in each condition were generated with high confidence (Supplementary Data File 1). Consequentially, AS isoforms considered absent in each condition may not be entirely absent but rather not expressed at a high enough level to be classified as present in this analysis. This PAV analysis resulted in the identification of AS isoforms with expression-level differences between spaceflight and ground control samples in both ecotypes and developmental stages tested (Fig. 1b). Orthogonal validation of AS isoforms counted as present or absent was performed by re-aligning the RNA sequencing reads to the reference genome utilizing a separate alignment program. Sequencing depth and splice junction coverage were then assessed using Integrative Genomics Viewer^14^ to experimentally validate a subset of the isoforms counted as present or absent in each condition (Supplementary Fig. 1–4). PAV analysis therefore enabled the identification of AS isoforms with expression-level differences in spaceflight, which reflects behavior of the transcriptional machinery within the cell.

Specific genes that undergo significant differential AS (dAS) were identified using CuffDiff^15^ to investigate whether decisions made by the splicing machinery differ in response to spaceflight. In both ecotypes and both developmental stages tested in this analysis, genes that undergo significant dAS between spaceflight and ground control samples were identified (Fig. 1c). Genes were identified as dAS if the isoform ratio differs significantly between the conditions examined (Supplementary Data File 2). A difference in isoform ratio between conditions is indicative of a difference in splicing decision between conditions, which is independent of transcription-level differences.

Interestingly, fewer genes undergo dAS in the Col-0 ecotype background collectively as compared to WS, which is opposite of the pattern observed in the literature between these two ecotypes regarding differentially expressed genes.^2^ Comparison of the genes that undergo dAS in either the Col-0 or the WS background reveals very little overlap in the identity of the genes alternatively spliced in response to spaceflight (Fig. 1d). Furthermore, gene ontology analysis does not reveal any obvious conclusions regarding the function or localization of the genes undergoing AS during spaceflight exposure.

**Fig. 2:**
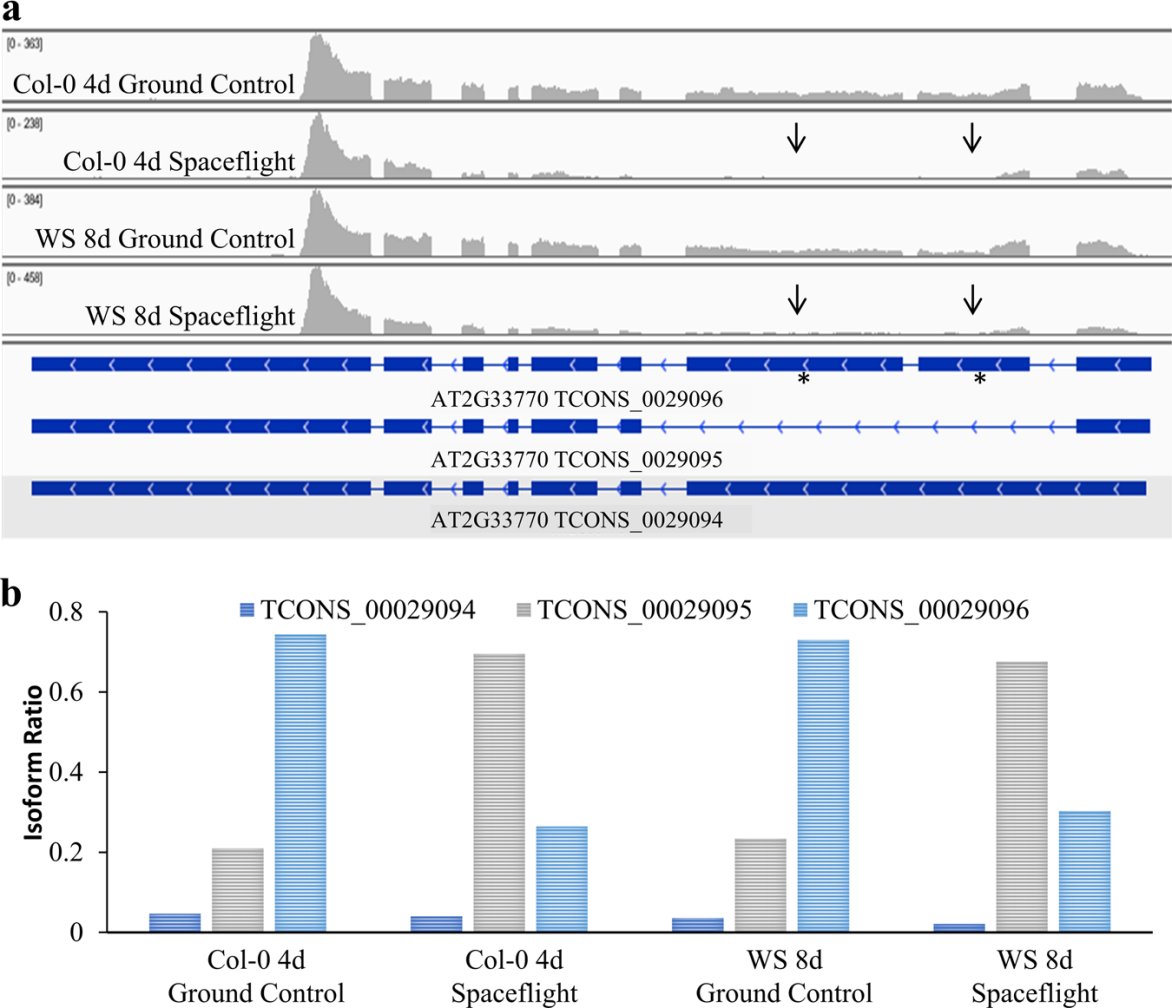
Visualization of switch in isoform ratio between spaceflight and ground controls for gene AT2G33770. **a** Screenshot from Integrative Genomics Viewer^16^ displaying sequencing coverage spanning the AT2G33770 gene model in spaceflight and ground control samples from either the 4-day Columbia-0 (Col-0) or 8-day Wassilewskija (WS) backgrounds. Asterisks indicate exons within the gene model solely present in transcript isoform TCONS_0029096. Arrows indicate lack of sequencing coverage in spaceflight samples corresponding to the unique exons in TCONS_0029096. Small numbers in the upper left hand corner of each panel denote the range of the *y* axis scale for each condition. **b** Isoform ratios in spaceflight and ground controls for transcript isoforms TCONS_0029094, TCONS_0029095, and TCONS_0029096 associated with gene AT2G33770 in Col-0 4-day and WS 8-day backgrounds. Isoform ratio was calculated by FPKM (fragments per kilobase of transcript per million mapped reads)

An example of dAS between spaceflight and ground controls in both Col-0 and WS is presented as an Integrative Genomics Viewer^14^ image that visualizes differences in sequencing coverage between AS isoforms of the gene AT2G33770 (Fig. 2a). This gene locus produces three AS transcript isoforms that utilize the same transcription start site—TCONS_00029094, TCONS_00029095, and TCONS_00029096. One AS isoform, TCONS_00029094, is only very lowly expressed in all conditions tested. However, AS isoform TCONS_00029095 is more common in spaceflight samples, whereas AS isoform TCONS_00029096 is more common in ground control samples (Fig. 2a, b). This example highlights a difference in pre-mRNA processing in response to spaceflight—a spliceosome-specific behavior as opposed to a difference in transcription.

This study represents the first observation of transcriptome-wide spaceflight-induced AS not only in plants but also in any living organism yet exposed to microgravity. The results presented here serve as strong evidence that AS has a role during the molecular adaptation process to the spaceflight environment in higher eukaryotic organisms such as plants and provides methods that can be used to identify AS isoforms from RNA sequencing datasets generated from other spaceflight-exposed organisms. However, the full role of AS during spaceflight adaptation must still be investigated by examining changes in the abundance of AS isoforms present in both conditions, the types of AS events occurring in spaceflight, and the functional implications of AS isoforms present or absent in spaceflight. In addition, it would be interesting to analyze the overlap between spaceflight differentially expressed genes and spaceflight-induced AS isoforms. Dissecting the spaceflight-induced transcriptome with both gene- and AS isoform-level resolution will elucidate better understanding of the molecular mechanisms leveraged by plants to adapt to the radically novel environment, that is, spaceflight.

## Methods

### Data acquisition—APEX03-2 spaceflight experiment

A subset of the data collected during the Advanced Plant Experiment 03-2 (APEX03-2) spaceflight experiment was utilized for this analysis. The APEX03-2 experimental methods have been previously described^16^ including detailed descriptions of the mission in which the experiment was launched, plant material preparations, and plant growth conditions both onboard the ISS and at KSC where ground controls were comparably grown. For this analysis, the data utilized comes from two wild-type accessions of *Arabidopsis thaliana* (Col-0 and WS) sampled at both 4 and 8 days of age during the experiment.

It should be noted that the APEX03-2 experiment is also referred to as “TAGES-ISA” (Transgenic Gene Expression System-Intracellular Signaling Architecture) by NASA operations nomenclature (http://www.nasa.gov/mission_pages/station/research/experiments/1059.html).

### Growth conditions and plant handling

Seeds sown on 10 cm^2^ petri plates were kept dormant during launch. Germination was initiated after transfer to the Vegetable Production System (VEGGIE) onboard the ISS or at KSC for ground controls. The plates were kept in vertical racks perpendicularly oriented to the light source during growth in VEGGIE. Lighting within the VEGGIE unit was set to provide between 100 and 135 µmoles/m^2^/s PAR (photosynthetic active radiation). Telemetric data collected on board the ISS during the spaceflight experiment were utilized to precisely mimic the environment onboard the ISS during the ground control experiment, carried out in the ISS Environmental Simulation Chamber at KSC. General methods associated with harvesting plant matter while on orbit for transcriptome analyses, plate de-integration once returned back to KSC, and transport back to the laboratory of the Principal Investigators for analysis has been described in detail for previous spaceflight experiments.^4^

### RNA extraction and sequencing

Four biological replicates were analyzed for each condition tested in the APEX03-2 experiment, where one replicate corresponds to the 10–15 plants grown on one petri plate. The plant material constituting each replicate was then subdivided for use in multiple downstream applications. Five to eight individual plants from each replicate of 4-day-old seedlings were combined for RNA extraction. However, to ensure that similar RNA concentrations from replicates containing 8 day-old seedlings were obtained, only 2–3 individual plants from each replicate of 8 day-old seedlings were pooled for RNA extraction. An Olympus dissecting microscope was utilized to separate leaf and root samples, while hypocotyl regions were set aside. Total RNA was extracted from the dissected roots using the Qiashredder and RNAeasy™ kits from QIAGEN (QIAGEN Sciences, MD, USA). Remnant DNA was removed via an on-column digestion utilizing RNase Free DNase (QIAGEN GmbH, Hilden, Germany). RNA quality was analyzed using an Agilent 2100 Bioanalyzer (Agilent Technologies, Santa Clara, CA, USA). A cDNA library was produced using the CloneTech SMART-Seq V4 Ultra Low Input RNA Kit (Clonetech Laboratories, Inc, cat#: 634890). Illumina sequencing libraries were then prepared via the Illumina Nextera DNA Sample Preparation Kit (Cat#: FC-131-1024). Bioanalyzer and quantitative PCR were used to quantify the libraries produced. Pooled equimolar samples were then sequenced using the NextSeq500 instrument in five separate flow cells.

### AS bioinformatics pipeline and downstream processing

Quality control was done to remove low quality reads and bases with Trimmomatic^17^ using parameters TRAILING:3, SLIDINGWINDOW:4:20, MINLEN:20. Reads were aligned to the *A. thaliana* version 10 reference genome (accessed from https://genome.jgi.doe.gov) with Bowtie2 version 2.2.9^18^ with parameter –fr. Low quality alignments were filtered out by piping the Bowtie2 output into Samtools^19^ via samtools view –q 30 –b | samtools sort. Picard 2.5.0 (https://broadinstitute.github.io/picard/) was used with the parameter REMOVE_DUPLICATES=true to remove potential optical duplicates from the alignments.

Transcript assemblies were conducted for each sample using three separate transcript assembly programs. Cufflinks^15^ was used with parameters –library-type fr-firststrand –u -F 0.05–max-intron-length 12000–no-faux-reads utilizing the *A. thaliana* version 9 reference genome and *A. thaliana* version 10 genome annotation (accessed from https://genome.jgi.doe.gov) to guide transcript assembly. StringTie^20^ was used with parameters -f 0.05 -j 2 –rf using the same genome and annotation as Cufflinks to guide assemblies. Trinity^21^ was used in genome guided mode with parameters –genome_guided_bam–genome_guided_max_intron 12000–full_cleanup–SS_lib_type RF–min_contig_length 50. The assemblies from Cufflinks and Stringtie were merged using the Stringtie merge function for each sample using parameters -F 1 -f 0.05. PASA^22^ was used to reconcile this merged assembly and the assembly from Trinity using parameters -C –R -t 〈Trinity fasta output〉 –cufflinks_gtf 〈merged gtf file〉 -I 12000–ALT_SPLICE–ALIGNER gmap, blat.

The transcriptome assemblies generated by PASA for each sample were merged with Cuffmerge. The file produced by Cuffmerge was then reformatted to agree with the reference genome annotation format and then compared against the reference annotation using gffcompare (https://github.com/gpertea/gffcompare). This merged transcriptome represents all potential transcript isoforms expressed in all of the samples being studied, including both newly discovered isoforms and those previously annotated.

The abundance of transcripts in each sample was then quantified with the rsem-calculate-expression functionality within RSEM^23^ using default parameters. The FPKM values of each transcript from each sample were parsed from the RSEM output into a matrix. This matrix was used for downstream analysis of PAV of alternatively spliced transcript isoforms. All isoforms expressed from parent genes without a minimum of two unique transcript isoforms identified within the dataset were removed. This category of discarded isoforms represents isoforms that were not produced as a product of AS as identified by the pipeline used in this analysis. In addition, within each condition all isoforms expressed at an FPKM < 10 in at least one out of four replicates were removed from the analysis. Following filtering, lists of spaceflight present, ground control present, and commonly present AS isoforms were generated.

A second round of alignments was done with STAR^24^ version 2.5.2b using parameters –alignIntronMax 12000–outFilterMismatchNmax 8–sjdbOverhang 100–outSJfilterReads Unique–outSAMmultNmax 1–outFilterType BySJout to generate files for junction spanning alignments. These alignments were filtered for duplicate reads using Picard with the parameter REMOVE_DUPLICATES=true. STAR allows for the alignment of junction spanning reads, whereas RSEM utilizes Bowtie for alignments, which does not allow for the alignment of these reads. Sequencing depth and splice junction support for a subset of isoforms present or absent in each condition and ecotype was then visualized using Integrative Genomics Viewer^14^ (Supplementary Fig. 1–4).

The alignments from STAR were also used as inputs into Cuffdiff.^15^ Cuffdiff was run using this merged transcriptome as a reference gtf file to discover differential transcription start site usage between spaceflight and ground controls in both ecotypes and developmental stages tested in this analysis. The transcription start sites of isoforms exhibiting differential splicing were parsed from the splicing.diff file output from Cuffdiff for each condition being tested. The isoforms associated with the transcription start sites were parsed from the isoforms.fpkm_tracking Cuffdiff output file and the expression, in FPKM, of these isoforms was parsed from the isoforms.read_group_tracking Cuffdiff output file. The isoform ratios were then calculated using a Python script for isoforms encoded from the same gene and sharing a transcription start site. All isoforms demonstrating an isoform ratio <0.01 in both spaceflight and ground control samples within a given condition were removed, resulting in the generation of lists of genes and their associated isoforms that undergo differential AS in each condition tested (Supplementary Data File 2).

### Reporting Summary

Further information on research design is available in the Nature Research Reporting Summary linked to this article.

## Supplementary information

Supplemental Figures

Supplemental Data File 1

Supplemental Data File 2

reporting summary

## Data Availability

The RNA-seq data reported in this paper are available in GEO under number GSE95586 and in NASA’s GeneLab data repository (https://genelab-data.ndc.nasa.gov/genelab/, reference number GLDS-218).

## Supplementary information

**Supplementary information** accompanies the paper on the *npj Microgravity* website (10.1038/s41526-019-0070-7).
