# Supplementary material for: Spaceflight-induced alternative splicing during seedling development in Arabidopsis thaliana
Source: NPJ Microgravity. 2019 Apr 3;5:9. doi: 10.1038/s41526-019-0070-7 (PMC6447593; doi:10.1038/s41526-019-0070-7)
Supplement: Supplementary file 1 — Supplemental Figures [file 41526_2019_70_MOESM1_ESM.pdf]

Supplemental Figures 1-4:

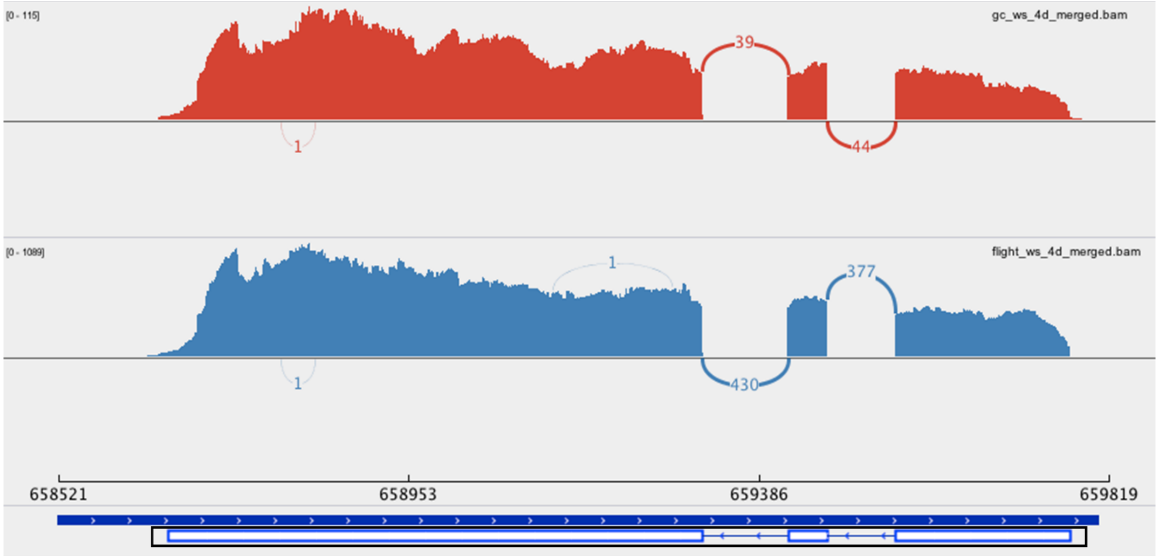

Figure S1. Splice Junction Support and Sequencing Read Depth for Isoform Transcripts of Genes Known to be Alternatively Spliced and Identified by PAV: Isoform TCONS_00010065 Associated with Parent Gene AT1G02920. The gene model corresponding to this isoform has been outlined in black, and exons contained within the isoform of interest have been colored white. TCONS_00010065 is present in spaceflight at 4 days of age in the WS ecotype background (blue) and absent in the comparable ground controls (red) by the criteria defined in this manuscript. Sequencing depth at each location along the gene model is represented by the height of the colored bars. Small numbers in the upper left hand corner of each panel denote the range of the Y-axis scale for each condition. The number of reads spanning each individual splice junction is displayed.

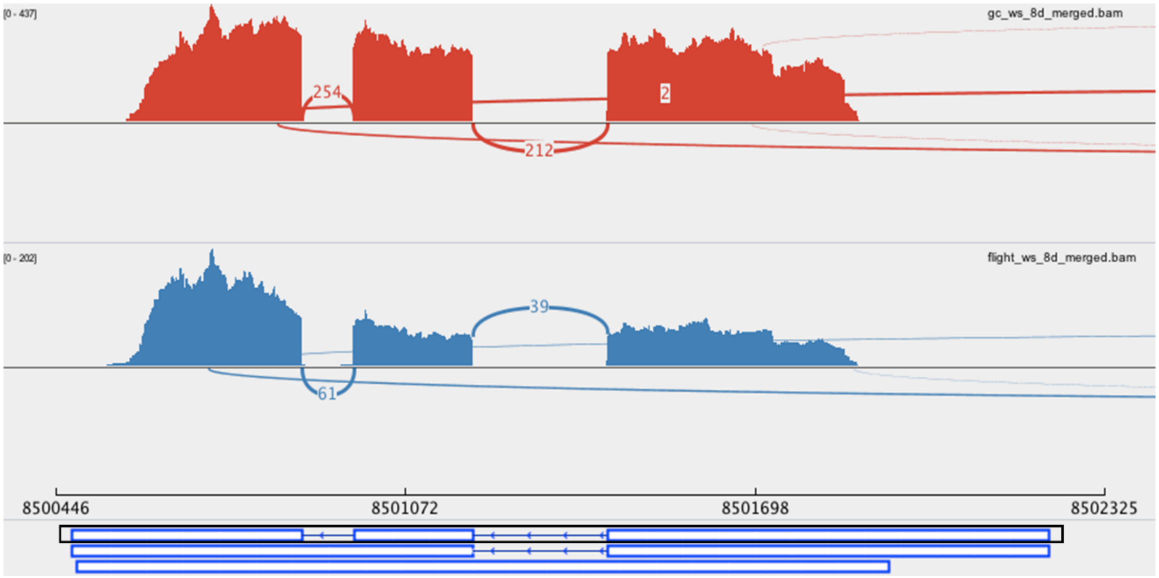

Figure S2. Splice Junction Support and Sequencing Read Depth for Isoform Transcripts of Genes Known to be Alternatively Spliced and Identified by PAV: TCONS_00069498 Associated with Parent Gene AT5G24770. The gene model corresponding to this isoform has been outlined in black, and exons contained within the isoform of interest have been colored white. TCONS_00069498 is absent in spaceflight at 8 days of age in the WS ecotype background (blue) and present in the comparable ground controls (red) by the criteria defined in this manuscript. Sequencing depth at each location along the gene model is represented by the height of the colored bars. Small numbers in the upper left hand corner of each panel denote the range of the Y-axis scale for each condition. The number of reads spanning each individual splice junction is displayed.

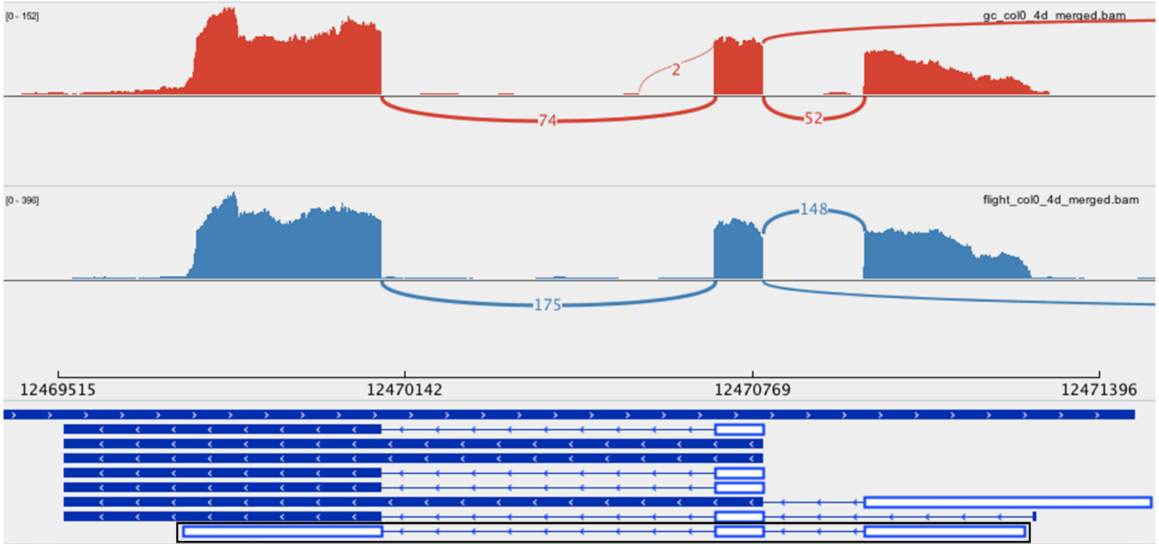

<u>Figure S3. Splice Junction Support and Sequencing Read Depth for Isoform Transcripts of Genes Known to be Alternatively Spliced and Identified by PAV: TCONS_00054704 Associated with Parent Gene AT4G24015.</u> The gene model corresponding to this isoform has been outlined in black, and exons contained within the isoform of interest have been colored white. TCONS_00054704 is present in spaceflight at 4 days of age in the Col-0 ecotype background (blue) and absent in the comparable ground controls (red) by the criteria defined in this manuscript. Sequencing depth at each location along the gene model is represented by the height of the colored bars. Small numbers in the upper left hand corner of each panel denote the range of the Y-axis scale for each condition. The number of reads spanning each individual splice junction is displayed.

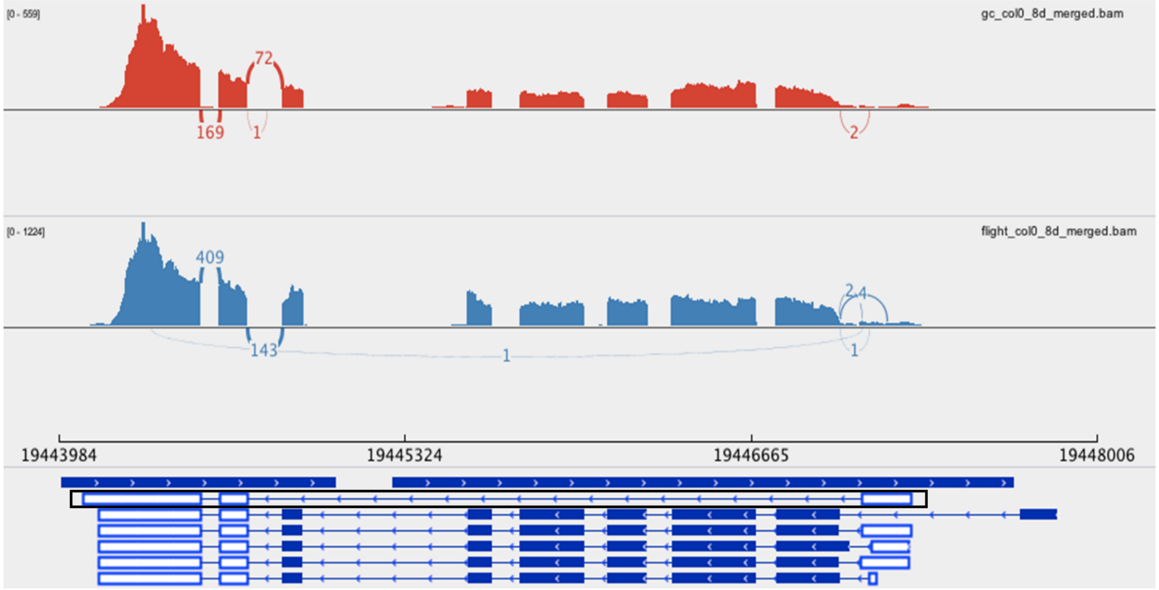

<u>Figure S4. Splice Junction Support and Sequencing Read Depth for Isoform Transcripts of Genes Known to be Alternatively Spliced and Identified by PAV: TCONS_00071815 Associated with Parent Gene AT5G48000.</u> The gene model corresponding to this isoform has been outlined in black, and exons contained within the isoform of interest have been colored white. TCONS_00071815 is present in spaceflight at 8 days of age in the Col-0 ecotype background (blue) and absent in the comparable ground controls (red) by the criteria defined in this manuscript. Sequencing depth at each location along the gene model is represented by the height of the colored bars. Small numbers in the upper left hand corner of each panel denote the range of the Y-axis scale for each condition. The number of reads spanning each individual splice junction is displayed.
